# First case of infective endocarditis due to NDM-type carbapenemase-producing Serratia marcescens in a preterm infant: a case report

**DOI:** 10.1099/acmi.0.000933.v3

**Published:** 2025-05-16

**Authors:** Zakaria Malihy, Ikram El Abdallaoui, Tilila Abassor, Salah Sghir, Yassine Ben Lahlou, Rachid Abilkassem, Elmostafa Benaissa, Mariama Chadli

**Affiliations:** 1 Department of Bacteriology, Mohammed V Military Teaching Hospital, Faculty of Medicine and Pharmacy (University Mohammed V), Rabat, Morocco; 2 Department of Pediatrics, Hospital of Children, University Hospital of Rabat, Rabat, Morocco; 3 Department of Pediatrics, Mohamed V Military Hospital of Rabat, Rabat, Morocco

**Keywords:** infective endocarditis, preterm infant, *Serratia marcescens*

## Abstract

*Serratia marcescens (S. marcescens)* is a Gram-negative rod-shaped bacterium belonging to the *Enterobacteriaceae* family, commonly found in various environments. This opportunistic pathogen can cause urinary tract infections, respiratory infections and septicaemia, but endocarditis is particularly rare and concerning due to its rapid and devastating progression. We report the first documented case worldwide of infective endocarditis (IE) caused by *S. marcescens* producing NDM-type carbapenemase, and the second reported case of *S. marcescens* endocarditis in a preterm infant. The patient was a preterm male infant born at 34 weeks of gestation, from a triplet pregnancy, admitted to the neonatal intensive care unit on day 2 of life for respiratory distress. The mother, aged 39, had undiagnosed gestational diabetes. Premature rupture of membranes had occurred 10 days before delivery, necessitating prophylactic treatment with amoxicillin. On day 4 of life, the newborn developed a fever with elevated C-reactive protein (CRP) levels and leucocytosis, leading to antibiotic therapy with colistin, imipenem and amikacin. Blood cultures revealed the presence of carbapenemase-producing *S. marcescens* sensitive to fluoroquinolones. A cardiac ultrasound showed a vegetation on the mitral valve, confirming the diagnosis of IE. Despite intensive treatment, the newborn died on day 16 of life due to septic shock. This rare case of endocarditis caused by *S. marcescens* highlights the severity of this infection in preterm infants. Treatment relies on appropriate antibiotic therapy. Prevention requires strict hygiene measures. Further research is needed to establish optimal therapeutic recommendations.

## Data Summary

No data were reused or generated in this study.

## Introduction

*Serratia marcescens (S. marcescens*) is a Gram-negative rod-shaped bacterium belonging to the *Enterobacteriaceae* family, commonly found in various environments, including water, soil, and plants. It is an opportunistic pathogen responsible for healthcare-associated infections. *S. marcescens* can cause urinary tract infections, respiratory infections, and septicaemia. Infective endocarditis (IE) caused by *S. marcescens* is exceedingly rare, accounting for ~0.14% of reported cases, yet it is particularly concerning due to its rapid and devastating progression [1]. The natural resistance of this bacterium to many antibiotics, along with its ability to form biofilms, significantly complicates the treatment of this infection [2].

IE is an inflammation of the endothelial tissue lining the heart chambers, usually of bacterial origin. This condition represents a medical emergency due to its potential to cause severe complications. The most commonly involved pathogens in endocarditis are staphylococci, streptococci and enterococci [3].

We report the first documented case worldwide of IE caused by *S. marcescens* producing NDM-type carbapenemase, and the second reported case of *S. marcescens* endocarditis in a preterm infant, highlighting the diagnostic and therapeutic challenges associated with this infection.

## Case report

The patient was a male preterm infant born at 34 weeks of gestation from a triplet pregnancy, admitted on day 2 of life to the neonatal intensive care unit (NICU) for respiratory distress. The 39-year-old mother had a history of cholecystectomy in 2022 and had experienced three pregnancies with five births (G3P5), as well as undiagnosed gestational diabetes discovered upon admission. Premature rupture of membranes had occurred 10 days before delivery, justifying prophylactic treatment with amoxicillin (1 g/8 h for 7 days) after clinical and biological exclusion of bacterial colonization or infection. Delivery was performed by caesarean section under spinal anaesthesia. The amniotic fluid was clear.

At birth, the newborn measured 46 cm, weighed 1.925 kg and had a head circumference of 31 cm. The Apgar score was 10 out of 10 at 1 min after birth, and his temperature was 37 °C. On day 2 of life, the preterm infant developed respiratory distress with metabolic acidosis, leading to his transfer to the NICU, where he received continuous positive airway pressure ventilation, which was weaned on day 4 of life, as well as hydration via peripheral intravenous access.

On day 4 of life, the preterm infant presented with fever associated with elevated CRP levels (from 3 to 242 mg l^−1^), leucocytosis (11 G l^−1^) with neutrophilia (7.8 G l^−1^) and normochromic normocytic anaemia (Hb at 8.8 g dl^−1^), justifying empirical antibiotic therapy with colistin, imipenem and amikacin.

Given the clinical presentation, 3 ml of blood was collected by direct venipuncture and inoculated into one paediatric blood culture bottle (BD BACTEC^™^ Peds Plus/F, Becton Dickinson) and then sent to our laboratory for bacteriological analysis. The blood culture bottle was incubated at 37 °C with continuous agitation in a BD BACTEC^™^ FX system. Bacterial metabolism within the bottle was detected by the automated system after 7 h and 13 min. Direct examination with Gram staining from the positive bottle revealed numerous Gram-negative bacilli. Further subcultures were performed on blood agar, blood agar with inhibitors (nalidixic acid-colistin), Polyvitex chocolate agar and chromogenic agar (CHROMagar), as well as 1:50-diluted broth flooded on Mueller–Hinton agar for antibiotic susceptibility testing using the disc diffusion method according to the 2024 recommendations of the Antibiogram Committee of the French Society of Microbiology. All subcultures were incubated at 37 °C in a CO_2_-enriched atmosphere of 10%.

After 24 h of incubation, except for the agar with inhibitors, which remained sterile, all subcultures showed a monomorphic appearance with numerous white, moist and shiny colonies. Analysis by MALDI-TOF MS (VITEK MS system, bioMérieux, Marcy l'Etoile, France) identified the species as *S. marcescens*.

Antibiotic susceptibility testing was confirmed by the microdilution method in a liquid medium and interpreted according to the 2024 recommendations of the European Committee on Antimicrobial Susceptibility Testing. Indeed, this strain exhibited multidrug resistance. The susceptibility profile with MIC is shown in Table 1.

**Table 1.** Susceptibility profile of the *S. marcescens* strain

Antibiotic	MIC (mg l^−1^)	Categorization
Ticarcillin/clavulanic acid	64/2	Resistant
Piperacillin/tazobactam	32/4	Resistant
Cefotaxime	32	Resistant
Ceftazidime	16	Resistant
Cefepime	8	Resistant
Imipenem	8	Resistant
Meropenem	8	Resistant
Ertapenem	4	Resistant
Amikacin	32	Resistant
Gentamicin	8	Resistant
Tobramycin	8	Resistant
Colistin	4	Resistant
Aztreonam	1	Sensitive
Levofloxacin	0.5	Sensitive
Ciprofloxacin	0.25	Sensitive
Tigecycline	0.25	Sensitive
Sulphamethoxazole-trimethoprim	0.5/9.5	Sensitive
Fosfomycin	32	Sensitive

From the isolated colonies, molecular biology analysis mediated by real-time nested RT-PCR of resistance genes coding for various carbapenemases (CARBA-R©, Cepheid, Sunnyvale, CA) returned positive with the detection of the *bla* New Delhi metallo-*β*-lactamase (*bla_NDM_*) gene. Ciprofloxacin treatment was initiated, and the initial antibiotic therapy was discontinued.

A urine sample collected by a collection bag, as well as cerebrospinal fluid obtained by lumbar puncture, was sent to our laboratory for cytobacteriological analysis. Both samples were sterile with white blood cell counts below their respective thresholds.

On days 5 and 6, blood cultures from two vials processed under identical conditions yielded the same strain of *S. marcescens*.

**Fig. 1.**
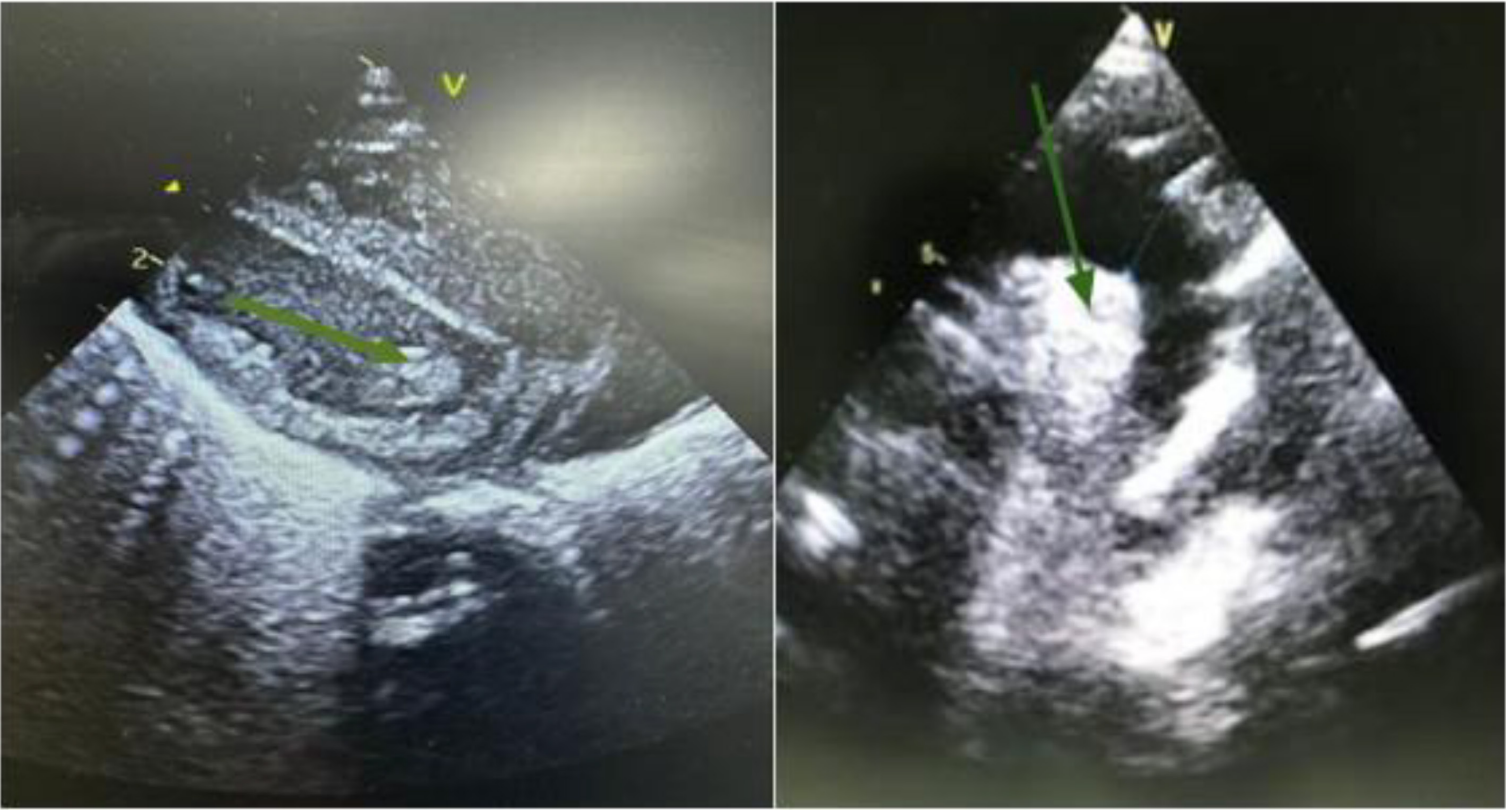
Echocardiographic image demonstrating a mitral valve vegetation (green arrow) in the setting of IE. The irregular, mobile mass is attached to the mitral valve leaflets, causing obstruction and regurgitation. This finding is characteristic of bacterial infection involving the heart valves.

Despite antibiotic treatment, the preterm infant’s respiratory distress worsened on day 8 of life, with the onset of haemodynamic instability requiring intubation and intensification of antibiotic treatment. A transthoracic echocardiogram showed a vegetation on the mitral valve (Fig. 1). Cardiac auscultation revealed a systolic murmur. According to the updated modified Duke criteria [4], the diagnosis of definite endocarditis was made based on persistent positive blood cultures for the same organism, echocardiographic findings and the appearance of a new heart murmur.

Despite intensive antibiotic therapy, the newborn did not improve and died on day 16 of life from septic shock with multiple organ failure.

## Discussion

*S. marcescens* is an opportunistic pathogen that can cause significant hospital outbreaks, particularly in neonatal units [5,7]. Preterm infants are especially susceptible to infections and colonization due to the immaturity of their immune systems. The risk of infectious complications is further exacerbated by other high-risk pregnancy factors, such as gestational diabetes and multiple pregnancies [8,9]. Our case exhibited all the aforementioned risk factors for infection.

Premature rupture of membranes is a common complication in multiple pregnancies [10]. Prophylactic antibiotic treatment, usually with amoxicillin, is used to prevent progression to chorioamnionitis. However, this treatment can lead to the selection of antibiotic-resistant pathogenic bacteria, which may cause massive maternal-foetal colonization [11]. Amongst the hospital-acquired bacteria naturally resistant to amoxicillin are *Enterobacteriaceae* (e.g. *Klebsiella pneumoniae*, *S. marcescens* and *Enterobacter cloacae*) and non-fermenting bacilli (e.g. *Pseudomonas aeruginosa* and *Acinetobacter baumannii*). Additionally, the overuse of broad-spectrum antibiotics like colistin creates selective pressure that favours the emergence of infections caused by *S. marcescens*, which is naturally resistant to this drug.

To the best of our knowledge, this is the second reported case of IE due to *S. marcescens* in a preterm infant and the first reported in Morocco. According to the literature, the rare cases of *S. marcescens* endocarditis typically occur in adults, particularly in intravenous drug users [12]. There are three published paediatric cases of IE caused by *Serratia* sp. with only one case involving *S. marcescens* in a preterm infant [13]. The other cases involved a 7-year-old burn victim infected with *S. marcescens* and a preterm infant who underwent cardiac surgery complicated by IE due to *Serratia liquefaciens* [14,15].

According to the literature [4], the presence of a venous catheter, bacteraemia caused by *S. marcescens* and the absence of a primary infectious focus suggest that the bacteraemia was likely catheter-related. The appearance of new valvular regurgitation, the presence of mitral vegetation on echocardiography and two positive blood cultures for *S. marcescens* taken 12 h apart confirm a definite endocarditis diagnosis based on the updated modified Duke criteria [4]. In our case, the occurrence of endocarditis can be explained by the persistence of bacteraemia, associated with the immaturity of the newborn’s cardiac endothelium, in the absence of identified cardiac lesions or malformations. *S. marcescens* IE can also involve the tricuspid valve [16].

*S. marcescens* possesses an inducible, low-level chromosomal cephalosporinase of the Amp-C type, which provides natural resistance to aminopenicillins, first-generation cephalosporins and aminopenicillin/inhibitor combinations. Treatment with beta-lactams, particularly clavulanic acid, cefoxitin and imipenem, can induce overproduction of this enzyme, leading to resistance to penicillins and cephalosporins [17]. In our patient, the reduced diameter around the imipenem disc suggested the following possible mechanisms: AmpC hyperproduction combined with either decreased outer membrane permeability or increased efflux pump activity or the production of a carbapenemase. Molecular analysis confirmed the production of an NDM-type metallo-beta-lactamase. This enzyme is a class B carbapenemase according to the Ambler classification. In *Enterobacteriaceae*, the genes encoding this enzyme are primarily associated with IncX3 plasmids, which can easily be transferred from one bacterium to another [18,19]. These plasmids often carry other resistance genes, conferring pan-resistance to antibiotics [20].

Currently, there is no consensus on the treatment of IE caused by *S. marcescens*. Several agents have been tested with variable results, including cefepime, piperacillin/tazobactam and fluoroquinolones [21]. According to the IDSA 2022 [22], newly developed antimicrobial agents (such as ceftazidime/avibactam or Cefiderocol) may be effective in the treatment of carbapenem-resistant *Enterobacterales*, although data remain limited for this specific indication and paediatric population

In our case, high-dose fluoroquinolone therapy proved ineffective. In the absence of fosfomycin and newer antimicrobial agents, infection with a multidrug-resistant *S. marcescens* strain represents a therapeutic dead end, significantly complicating patient management.

Several studies have highlighted the effectiveness of reinforced hand hygiene, contact precautions and environmental decontamination in halting NICU outbreaks [23,24]. Infection control measures are essential in preventing *S. marcescens* transmission, particularly in NICUs, where the pathogen has been implicated in numerous outbreaks.

## Conclusion

This rare case of IE caused by *S. marcescens* underscores the danger posed by this opportunistic pathogen to vulnerable individuals and its rapidly disastrous progression. Early identification and prompt initiation of targeted antimicrobial therapy are crucial in managing neonatal sepsis, given its rapid progression and high mortality rates. Treatment relies on bactericidal antibiotic therapy. Further studies are needed to establish therapeutic guidelines. This infection is preventable through the adoption of strict hygiene measures. Implementing stringent infection control measures, such as hand hygiene and the use of personal protective equipment, is essential to prevent the spread of multidrug-resistant organisms in NICUs.

## Abbreviations

CRP: C-reactive protein
IE: infective endocarditis
NDM: New Delhi metallo-β-lactamase
NICU: neonatal intensive care unit
